# Nutrition, Lifestyle, and Environmental Exposures in Endometriosis: Toward Personalized Supportive Care

**DOI:** 10.3390/nu18193252

**Published:** 2026-10-02

**Authors:** Antonella Iaconis, Marika Cordaro

**Affiliations:** 1 Department of Chemical, Biological, Pharmaceutical, and Environmental Sciences, University of Messina, 98166 Messina, Italy; antonella.iaconis@studenti.unime.it; 2 Department of Biomedical, Dental and Morphological and Functional Imaging, University of Messina, 98125 Messina, Italy

Endometriosis is a chronic, estrogen-dependent inflammatory disease primarily associated with pelvic pain, infertility, and fatigue, as well as gastrointestinal symptoms, resulting in a significant reduction in quality of life. Its clinical presentation is extremely variable, and the mechanisms underlying the lesions and symptoms involve hormonal signaling, immune dysfunction, oxidative stress, neuroinflammation, angiogenesis, and fibrosis [1,2]. Hormonal treatment, analgesia, and surgery remain central to management, yet none provides a universally effective or definitive solution. This therapeutic gap helps explain the growing interest in diet and lifestyle as supportive strategies, particularly among patients seeking options that can be integrated into long-term care.

It is worth noting that current clinical guidelines adopt a cautious approach to non-pharmacological interventions, while recent literature reviews conclude that available evidence remains insufficient to recommend universal dietary regimens. Nutritional research on endometriosis faces several challenges: heterogeneous phenotypes, small sample sizes, self-reported dietary intake data, inconsistent outcome measures, and the difficulty of distinguishing effects on symptoms from effects on lesion biology. Furthermore, restrictive diets may entail nutritional and psychological risks if adopted without clear indications [3,4]. Research must now determine which nutritional or lifestyle measures may offer safe, measurable benefit to defined patient groups while established therapy remains in place.

This Special Issue brings together five review articles that examine this question from complementary perspectives. The collection moves from whole-diet and lifestyle approaches to dietary fiber and antioxidant supplementation before considering toxic elements as environmental exposures whose internal dose may itself be modified by nutritional status. These contributions describe a shared biological framework centered on estrogen metabolism, the gut microbiota, inflammation and redox homeostasis. They also reveal a consistent gap between mechanistic plausibility and evidence from well-controlled clinical interventions.

Szczuko et al. (contribution 1) provide a systematic overview of dietary strategies and food elimination approaches in endometriosis. Their analysis addresses anti-inflammatory patterns, Mediterranean and MIND diets, low-FODMAP, gluten-free and low-nickel approaches, dietary fat, red meat, dairy products, fiber and antioxidant-rich foods. From a clinical perspective, no single elimination diet can be universally recommended. An anti-inflammatory dietary pattern may represent a reasonable initial approach, whereas additional dietary restrictions should be considered only in the presence of documented food allergy, intolerance, or a relevant gastrointestinal phenotype. This individualized approach is particularly important, as symptomatic improvement following dietary modification does not necessarily indicate an effect on the underlying disease mechanisms.

Boroncsok et al. (contribution 2) broaden the discussion beyond diet to include physical activity, sleep, stress, body weight, smoking, alcohol, and exposure to endocrine-disrupting chemicals. Mediterranean-style and low-inflammatory dietary patterns emerge as practical candidates for supportive care, while regular exercise may contribute to pain control, metabolic health, and psychological well-being. The available evidence also supports considering lifestyle interventions as complementary, rather than alternative, strategies to pharmacological and surgical management. Interpretation of the reported benefits remains limited by the predominance of observational studies, heterogeneous intervention designs, and the frequent combination of multiple lifestyle components within the same protocol. Future well-designed trials should therefore clarify the contribution of individual interventions, identify the patient subgroups most likely to benefit, and determine the duration required to achieve clinically meaningful effects.

Czerniak et al. (contribution 3) focus on dietary fiber as a link among estrogen metabolism, the gut microbiota and immune regulation. Fiber may alter enterohepatic estrogen cycling, support microbial production of short-chain fatty acids, strengthen intestinal barrier function, and reduce inflammatory signaling. These mechanisms provide a biological rationale for the potential relevance of fiber-rich foods in estrogen-dependent inflammatory disorders. Observational studies suggest that higher fiber intake may be associated with a reduced symptom burden, although randomized trials specifically evaluating fiber remain limited. The clinical effects of fiber may also depend on its source, type, and dose, particularly in patients with bloating, irritable bowel syndrome, or other gastrointestinal comorbidities. Accordingly, any increase in fiber intake should be introduced gradually, preferably through whole foods and tailored to individual gastrointestinal tolerance.

Pokorska-Niewiada et al. (contribution 4) critically examine non-mineral antioxidants in relation to the redox disturbances observed in endometriosis. Oxidative stress can amplify inflammatory signaling, mitochondrial dysfunction, nociception and lesion persistence, providing a sound rationale for redox-active interventions. However, clinical support is inconsistent. Melatonin has the most consistent evidence for a moderate reduction in pain and *N*-acetylcysteine remains promising, whereas findings for vitamins C, E, and D and coenzyme Q10 are insufficient or inconsistent. The review appropriately separates biological rationale from therapeutic efficacy: antioxidant supplements should not be presented as a standalone treatment, and their place in care requires better randomized trials with clinically relevant endpoints.

On the other hand, Pokorska-Niewiada et al. (contribution 5) review cadmium, lead, mercury, and arsenic as possible environmental contributors to endometriosis through endocrine disruption, oxidative stress, chronic inflammation, and epigenetic alteration. Cadmium currently has the strongest, although still non-causal, human evidence. A particularly useful insight is that biomonitoring values cannot be interpreted independently of nutritional status. Iron deficiency may increase intestinal cadmium absorption through shared transport pathways, potentially raising internal exposure even when external exposure is similar. This interaction connects environmental health with clinical nutrition and supports the inclusion of iron status, exposure sources and standardized biological matrices in future studies.

Across the five contributions, personalization consistently emerges as both a clinical priority and a methodological necessity. A comprehensive nutritional assessment should account for disease phenotype, predominant symptoms, gastrointestinal comorbidities, habitual dietary patterns, supplement use, food allergies or intolerances, nutritional deficiencies and relevant environmental exposures. Future research should reflect this complexity through prospective, multicenter studies using standardized diagnostic and phenotypic criteria, clearly defined dietary interventions and harmonized core outcomes encompassing pain, quality of life, fertility, gastrointestinal symptoms, adverse events and adherence. Mechanistic biomarkers may provide valuable biological insight, but they should complement rather than substitute patient-centered outcomes. Adequate follow-up is also essential to distinguish transient symptom improvement from sustained and clinically meaningful benefit.

The collection supports a measured conclusion. Diet and lifestyle can form part of multidisciplinary endometriosis care, but current evidence favors supportive, individualized measures over universal elimination diets or supplement regimens. Fiber-rich and Mediterranean-style dietary patterns are plausible low-risk options when adapted to gastrointestinal tolerance, while antioxidant supplements require more selective use and stronger clinical confirmation. Environmental exposure assessment adds another dimension, especially where nutritional status modifies toxicant uptake. As Guest Editors, we thank the authors for their contributions, the reviewers for their careful evaluations, and the editorial team of *Nutrients* for its support. We hope this Special Issue encourages studies capable of translating mechanistic insight into precise, safe, and clinically testable recommendations.
